# Buffalo Academy of Medicine

**Published:** 1901-04

**Authors:** F. W. McGuire


### Society Proceedings.

### BUFFALO ACADEMY OF MEDICINE.

*Section on Pathology, January 15, 1901.*

REPORTED BY F. W. McGUIRE, M. D., Secretary.

THE regular meeting was held at the Academy rooms, Tuesday, January 15, 1901, Dr. EUGENE A. SMITH in the chair, twenty fellows being present.

The minutes of the previous meeting were read and approved.

Dr. GROVER W. WENDE presented a case of multiple pigmented sarcoma of the skin, cured by injections of arsenic every other day, commencing with 5 drops and increasing to 18, and continuing the treatment for a year and a half.

Dr. MARSHALL CLINTON presented a case of sarcoma occurring in a scar one year after removal of a lipoma. It is now six months after removal of the sarcomatous growth and there is no return.

The essayist of the evening was Dr. J. C. JOHNSON, assistant pathologist at Cornell University, who read a paper entitled,

### SARCOMA AND SARCOID GROWTHS OF THE SKIN.

Dr. Johnson divided the growths into three groups: (1) True sarcoma; (2) the leukemic group; (3) sarcoid growths, and taking each group he discussed them separately.

*First*—Sarcoma he divides into spindle-celled, round-celled and giant-celled or myeloid. These are sometimes pigmented. The spindle-celled are the most common. Some spindle-celled growths are rapidly fatal, while others, again, indistinguishable from these, microscopically, run a comparatively indolent course. Any swiftly growing sarcoma, however, especially if it contains autochthonous pigment, has the gravest prognosis. Encapsulation, on the other hand, improves the outlook, since it limits the extension and permits of removal.

He doubts that the spindle-celled variety arises exclusively from the adventitia of the vessels, and sees no reason why the connective tissue corpuscles are not capable of taking on malignant action. The myeloid form occurs in the skin only by extension from the periosteum.

In discussing treatment he mentioned surgical interference as about the only hope; this at times did damage, as when the tumor was not all removed and a more acute process would develop. He also mentioned Coley's toxin. He thinks the nonpigmented spindle-celled sarcoma offered the best chance for its success. Arsenic he thinks is of questionable utility, but is sometimes of advantage.

*Second*—He discussed the leukemic group. There he includes leukemia, pseudo-leukemia, malignant lymphoma, variously styled lympho-sarcoma, and small round-celled sarcoma. He pointed out how great a task it was to differentiate these growths, the blood count offering some value, especially where leukemia had reached its well marked stage of leucocytosis. Histologically they are very similar. Treatment offers very little hope; operations are useless; Coley's toxin is ineffectual. Arsenic is usually of no avail, but in some cases proved beneficial.

*Third*—Sarcoid growths, under which he considered granuloma fungetis, multiple idiopathic pigmented sarcoma, sarcomatosis cutis and multiple benign sarcoida. Granuloma fungetis is perhaps the most extraordinary disease to which the skin is subject. He divides its course into three periods: (1) Premycosic or prefungoid; (2) stage of infiltration; 3 that of tumor formation.

The treatment lies in holding the disease in check as much as possible and making patient comfortable. Arsenic holds a high place. He gave descriptions of each of the members of this group. The treatment was mostly injections of arsenic, which offers good results in some cases.

### DISCUSSION.

Dr. H. U. Williams thought Dr. Johnson should be congratulated on his very clear classification of sarcomatous growths of the skin. He said sarcoma was a dumping ground for growths; when it was not known what to call them they were usually called sarcoma. He thought it was a good arrangement to eliminate a certain number of sarcomatous growths by putting them in a group by themselves. He said some were malignant and some were not; the whole subject in general was most unsatisfactory. He has seen sarcomatous growths in dogs.

Dr. A. E. Diehl liked the author's simple classification, but thought that sarcoma was rare; he had only seen three cases. He said that leukemia and pseudo-leukemia were certainly not sarcomas.

Dr. Harvey R. Gaylord wished to express his satisfaction with the paper. He thought Dr. Johnson's classification was very clear and simple. He also thought that embryological growths could not be taken into consideration in the classification of sarcomatous growths, as we know little if anything about them. He has examined several specimens of sarcoma in animals.

Dr. M. Hartwig cited a case of Hodgkin's disease, which improved phthisis set in. The phthisis subsided and Hodgkin's disease returned more pronounced.

Dr. Johnson, in closing, said he was very much gratified at the discussion. He further remarked that the literature on sarcoma of skin was very unsatisfactory. He thought it was necessary to have the whole tumor when making an examination for sarcoma.

The next paper was by Dr. Grover W. Wende, entitled,

URTICARIA.

The author considers it one of the most interesting of diseases, because in many cases it can be evoked at will and a study of its pathological process can be made. In this connection he described the evolution and devolution of factitious urticaria, and went quite minutely into the physiological phenomena. On this he cited opinions of different authors and gave as his own opinion that it was caused by a stimulation or irritation of the plexiform arrangement in the skin itself.

He said that urticaria was not confined to the skin alone, but that it sometimes affects the mucous membrane, and cited Sir Andrew Clark's opinion regarding the phenomena of asthma. He then discussed some of its less known causes: hydateds, parturition, passage of uterine sound, pregnancy, menstruation, and the like. He then described unusual cases: (1) a case of urticaria hemorrhagia; (2) a very chronic case, which appeared to take its origin in chickenpox; (3) a case of urticaria pigmentosa; (4) a giant urticaria.

Treatment resolves itself into two points: (1) Discover cause and remove it; (2) mitigate the effects. Regarding causes we must not forget the possibility of local irritation as fleas, bugs, pediculi, irritating clothing, and the like. Regarding indirect irritants, as unusual articles of diet, the administration of a prompt cathartic or saline laxative will usually cure the disease. In chronic cases a saline laxative, mineral acids, rhubarb and soda, salol, resorcin and other intestinal antiseptics, are of service in many cases. In this class of cases the diet must be regulated, each case being a study of itself, probably a milk diet with otber articles added with care, will be most serviceable. Alcohol in all forms must be stopped.

If gouty or rheumatic, treatment for this condition must be adopted. If periodical, quinine may do good. Salicylates often prove beneficial, even when there is no evident rheumatic tendency. In neurotic cases, arsenic, bromides, belladonna, antipyrine or galvanism may be tried. In obstinate cases patient should be sent from home.

Local treatment, parts may be sponged with alkaline lotions; sometimes acid lotions will give relief, as vinegar and water, carbolic acid and vaseline, alcohol and water, menthol 1 to 10 per cent., chloral and camphor, one-half to one ounce rubbed together and added to starch or unguentum simplex. Baths with bran or bicarbonate of soda are frequently useful.

DISCUSSION.

Dr. A. E. DIEHL said that Dr. Wende had gone so thoroughly into the subject that very little was left for discussion. He reported a case that proved irrisistible to all medication which was cured by leaving the city, *i. e*, by change of environment.

Dr. JOHNSON believes that people have a constitutional predisposition to urticaria caused by increased coagulability of the blood. He believes icthyol to be absolutely useless in this disease.

The section adjourned at 11 p. m.

---

### *Section on Medicine, February 12, 1901.*

REPORTED BY JULIUS ULLMAN, M. D., Secretary.

The fifth regular meeting of the Section of Medicine, was held in the Library Building, Tuesday, February 12, 1901, Dr. ELI H. LONG presiding.

Drs. CHARLES CARY and JULIUS ULLMAN reported a case of psychic equivalent of epilepsy. The patient is a contractor, 38 years of age, and born in this country—New York City; the father died of a paralytic stroke at the age of 56; the mother died of what the patient termed "rheumatism of the heart." One brother when a child had fainting spells and many specialists in New York were consulted, but gave little hopes of cure. He is, however, now living and well. The patient suddenly lost all remembrance of self consciousness from Friday at noon in New York City, until he found himself in a private room of the Buffalo General Hospital the following Wednesday evening. The last remembered by him is that being worried about an invalid wife, and also being mentally exhausted as the result of active participation in a trying political campaign; he entered the elevated train in New York City to go to his home. During the time of the loss of his self-consciousness he undertook a railway journey from that city to Buffalo. (Lesegue and Legrand du Saulle reported a case where the patient took passage at Havre and did not come to himself until he reached Bombay.)

The patient was found by the police aimlessly wandering about the streets and sent to the hospital. During the time that he was in the hospital it was impossible to get any account from him. He repeatedly called for the "senator" and for his wife, but there was complete amnesia as to names or location. Wednesday night he regained consciousness and was much surprised to find himself in Buffalo. While driving last summer he struck his head and was unconscious for several hours, since which he talks a great deal in his sleep. On the grounds of this peculiar history the diagnosis was made and substantiated by Dr. James W. Putnam. Since the patient's return to New York City he experienced another slight attack three and one-half hours in duration, and is to undergo an operation for the relief of these attacks.

Reference was made in the paper to the case of Sadie McMullen, who was observed at the Buffalo State Hospital as a case of epileptic insanity and which was described July, 1891, in the *Journal of Nervous and Mental Diseases* as a medico-legal case of psychic equivalent.

Dr. J. W. Grosvenor read a paper, with a record of a case, entitled,

PEMPHIGUS.

A rare disease, characterised by appearance of blebs or bullæ on external surface of body and mucous membranes. Acute and chronic. Acute occurs both in children and adults; chronic exclusively in adults. Acute form contagious and epidemic. Most important chronic forms are P. vulgaris, P. vegetans and P. foliaceus. The basal cause of pemphigus not known with certainty. Quite probable that it is due to a microörganism. Gibier has found bacteria in contents of the blebs. Chronic forms in debilitated adults almost uniformly fatal. Recovery from P. vegetans and P. foliaceus is exceedingly rare. Children of vigorous constitution and healthy environment generally recover. Treatment consists of quinine and arsenic in large doses persistently followed and soothing powders locally. The author gave a record of a typical case of P. vulgaris in a woman 70 years of age. The case had a favorable issue after two years of treatment with quinine and arsenic internally, and borated talcum powder externally.

DISCUSSION.

Dr. Grover W. Wende demonstrated charts and plates of this disease and says the disease is an unusual one, and spoke of the confused state of the classification of this disease and the difference of opinion as to conditions regarded by Kaposi and his school and that of the American dermatologists.

Dr. Alfred Diehl spoke briefly of an outbreak of impetigo contagiosa as seen by him among babies and nurses at the Erie County Hospital.

Dr. Grosvenor closed the discussion.

Dr. Albert E. Woehnert read a paper

ON THE VALUE OF BLOOD EXAMINATIONS BASED ON FIVE HUNDRED EXAMINATIONS.

He said that the importance of blood examinations as a means of differential diagnosis in disease is of recent time. In counting blood the sources of error may be faulty technique or the daily variation in blood. The selection of a certain hour to make a blood count is important, for there always is a physiological leucocytosis after meals. The corpuscles should be counted in separate counters. In the writer's experience the hematokrit is not an accurate instrument to count the corpuscles. Most instruments for the estimation of hemoglobin are based on a color test and the results obtained depend, therefore, too much on personal equations. The best results have been obtained by using the specific gravity method of Hammerschlag. Fibrin is increased in inflammatory conditions. Leucocytosis can often be detected in unstained specimens.

The blood is diagnostic in pernicious anemia, chlorosis, malaria and leukemia. In twenty cases of pernicious anemia examined, the average number of reds were 1,342,270; whites, 4,241; hemoglobin, 34 per cent.; hemoglobin index, 125, and specific gravity between 1,040 and 1,052. There was macrocytosis and poikilocytosis with nucleated reds. Pernicious anemia must be differentiated from the secondary anemia of cancer.

*Chlorosis.*—Average number of reds, 4,434,000; whites, 7,157; hemoglobin, 43 3-4 per cent.; color index, 63 per cent. The red cells varied in size and shape and plainly showed the lack of hemoglobin.

*Leukemia.*—The blood is very characteristic; the reds are reduced, whites show an enormous leucocytosis, .the large proportion of myelocytes being very characteristic. The reds show many nucleated cells. The character of leucocytes in the lymphatic leukemia was mentioned and a case was reported in which the reds fell from 5,000,000 to 800,000 in three months and the whites were reduced from 34,000 to 600, due to a secondary and terminal infection. Secondary anemia is the result of known causes, and the study of the leucocytes is important in this form of anemia. Very often the presence of leucocytosis or a leucopenia is of diagnostic value. The red cells are not so much disturbed in size and shape as in the essential anemias, and hemoglobin reduction closely follows the reduction of the red cells.

The anemia of malignant disease was described. Blood examinations are of great value in appendicitis. In fifteen cases there was a leucocytosis ranging from 10,000 to 25,000 and in all cases over 15,000 pus was found.

The blood of syphilis, tuberculosis and malaria, was described. The prognostic value of blood in pneumonia was spoken of; an eosinophilia, showing a post critical stage; so also the increase in eosinophiles in trichinosis and the inflammations of the skin and mucous membranes.

DISCUSSION.

Dr. I. P. Lyon insists on the hour of examination; after a meal there may be a variation of 33 per cent. It is well therefore to examine before meals. He believes that blood examinations are of the utmost importance and that the adjuvant to diagnosis has come to stay. In very severe forms of pneumonia or appendicitis, there is not much leucocytosis, and this may be regarded as a sign fatal to prognosis. He referred to a differentiation between an inflammatory condition of kidney—pyelonephritis or surgical kidney—in which there is a marked leucocytosis and fibrin increase, whereas in malignant disease of the kidney there is leucocytosis without the increase of fibrin.

Dr. Lyon also referred to a case where he found a leucotysosis of 30,000, and which on operation proved to be a perinephritic abscess.

Dr. A. L. Benedict uses the centrifuge and hematokrit for purposes of estimating the number of reds and whites, and in his experience this method is easy and quite accurate. He does not believe that the specific gravity of blood has more to do with hemoglobin than the specific gravity of urine has to do with the estimation of urea. He spoke of the methods used by him to fix slides. According to the ordinary stains bat's and rat's blood don't stain. He believes that we will soon have to investigate the blood plasma and chemistry of the blood.

Dr. WOEHNERT closed the discussion.

---

*Section on Ophthalmology, Otology, Rhinology and Laryngology, February 18, 1901.*

REPORTED BY GEORGE F. COTT, M. D., Secretary.

Sixth meeting, Monday, February 18, 1901. Dr. FRANK WHITEHILL HINKEL in the chair. The chairman called the meeting to order and Dr. MILLENER, secretary pro tem., read the minutes of the previous meeting, which were approved.

Dr. J. C. CLEMESHA presented a case of

MACULAR CHOROIDITIS WITH CHOLESTERIN CRYSTALS.

DISCUSSION.

Dr. BLAAUW.—The case Dr. Clemesha presents, I consider to be retinitis hemorrhagica. I would like to ask Dr. Clemesha why he calls it choroid retinitis? The white plaques do not say anything for an affection of the choroid; they are frequently met in other forms of retinitis. He said further that it was a rather peculiar picture of the choroide state with obscure pigment. He never saw such an affection.

Dr. BENNETT agreed with Dr. Blaauw.

Dr. Hinkel asked whether any such lesion was noticed following influenza.

Dr. WILLIAM C. KRAUSS then read a paper on

THE CLOSE RELATIONSHIP BETWEEN THE EYE AND NERVOUS DISEASES.

This close relationship is due to the following reasons: (1) Because of the close continuity of the two organs; (2) the eye receives a large number of important cranial nerves, anatomically extending from the cephalic border of the chiasma, to beyond the caudal border of the Pons, an area equal to one half the basilar surface of the brain; (3) the size and expansion of the optic nerve into the retina which reflects by its complex structure many of the disorders of the brain; (4) the varied physiology of these nerves being motor, sensory, trophic and special sense in function; (5) the intimate connection between the arterial and lymphatic supplies of both eye and brain.

Nervous diseases are expressed in the eyelids, either by (1) paralysis of levator palpebræ superioris, producing ptosis; (2) paralysis of the oricularis palpebrarum, producing lagophthalmos; (3) spasm, and (4) trophic disturbances.

Paralysis producing ptosis may be due to (1) congenital disturbances; (2) oculo-motorius disease; (3) apoplexy into the angular gyrus; (4) brain syphilis; (5) reflex disturbance; (6) sympathetic nerve disease; (7) locomotor ataxia; (8) hysteria; (9) basilar disease; (10) corpora quadrigemina disease; (11) crus cerebri disease, and (12) asthenic bulbar paralysis.

Paralysis producing a lagophthalmus is found in Bell's palsy and diplegia facialis. Spasm of the eyelids is met with in (1) blepharospasm; (2)spasm nictitans; (3) tic convulsif; (4) exophthalmic goitre, as Von Graefe's sign and Stelwag's sign.

Trophic disturbances are found in myxedema, angio-neurotic edema and acromegaly. The orbit in nervous diseases is expressed either by abnormality in size, as (1) microphthalmus and (2) macrophthalmus, both being congenital and significant of degeneracy, (3) enophthalmus and (4) exophthalmus. Paralysis of the orbital muscles producing strabismus is found in disease of the 3d, 4th and 6th nerves, ophthalmoplegia, locomotor ataxia and exophthalmic goitre. Spasm of the orbit producing nystagmus is found in disseminated sclerosis, Friedreich's ataxia and syringomyelia.

The pupils, disc, retina and vision were briefly considered and the following conclusions drawn regarding the relationship between choked disc or optic neuritis and brain tumors: (1) Optic neuritis is present in about 90 per cent. of all cases of brain tumor; (2) it is more often present in cerebral than in cerebellar cases; (3) the location of the tumor exerts little influence over the appearance of the papillitis; (4) the size and nature of the tumor exert but little influence over the production of the papillitis; (5) tumors of slow growth are less inclined to be accompanied with optic neuritis than those of rapid growth; (6) it is probable that unilateral choked disc is indicative of disease in the hemisphere corresponding to the eye involved; (7) it is doubtful whether increased intracranial pressure is solely and alone responsible for the production of an optic neuritis in cases of brain tumor.

DISCUSSION.

Dr. Blaauw.—Papers are of two kinds: First, the kind that must be read beforehand in order to be able to discuss it properly; second, that which we can hear and discuss immediately afterward. Dr. Krauss's paper is of the first kind. Notwithstanding this, I will say a few words to show my appreciation.

The Doctor says in his paper, "the diagnosis of hysteria is easy." I do not agree with him. In the early stages of tabes, for instance, we find ptosis or a paralysis representing paresis of one of the external ocular muscles, which improves without treatment, but may or may not relapse. How often has the diagnosis of hysteria not been made in such cases? It is the same in the early stage of multiple sclerosis.

From another standpoint the question of ptosis is of interest. Formerly we used to diagnosticate a nuclear affection as soon as a single or several external ocular muscles, innervated by a common nerve, became paralyzed. The history of ptosis shows us that we have no right to do this, because in ptosis we might find a vascular affection. I do believe that monocular spasms of the eyelids are the result of no local ocular cause, but may be the result of a cerebral affection.

As to the third class of cases referred to in this paper, I think that the diagnosis of neuritis must be made in the direct image. We accept, with Gowers, three stages of neuritis; the first stage can only be made out in the direct image. Still we must always keep in mind the physiological appearance where the borders of the pupil are so indistinct that we would make diagnosis of neuritis in the reversed image. That we should find more optic neuritis in cerebral tumors than in cerebellar ones, is the first time I ever heard it mentioned. I can not accept the statement just yet. We have not the right at present to call the symptoms of optic neuritis, three to six diopters, pathognomonic since it has been found in anemia, of which possibility some thirteen cases have now been reported.

Dr. Bennett.—I think many of the cases of spasm of the orbicularis are due to errors of balance of the vertical recti. One case I can recall, which was immediately and absolutely cured by a tenotomy of the right superior rectus, relieving four degrees of hyperphoria. This case has remained perfectly well for fifteen months without anything further being done. As to the question of brain tumor, I have often been astonished at the duration of life after diagnosis has been made. I can recall two cases. The first, a school girl, in which the diagnosis of brain tumor was made nine years before death, by Dr. Park Lewis, who found a papillitis on one side. The sight of the other eye remained good for six years, then began to fail. She then came to the dispensary, where I found an optic atrophy in the eye first affected, and a very marked optic neuritis in the other. She gradually became totally blind but lived for three years after. At the autopsy, the eye first affected was firmly adherent to a tumor surrounding the optic nerve and many other tumors were found in other portions of the brain. Dr. Earl Lothrop found the tumors to be sarcomatous.

The second case: Five or six years ago I examined a girl and found double optic neuritis, facial paralysis of the right side and uncertain gait. The exact details of the case escape me at the present moment. I made a diagnosis of brain tumor and gave a bad prognosis. The family wished further advice, so Dr. Crego was called in consultation. He confirmed my diagnosis and gave the patient but a few months to live. She is alive today, but is totally blind.

Dr. Burrows.—Can Dr. Krauss throw some light on the obscure cases of double optic neuritis?

Two cases at the General Hospital had double optic neuritis, one with complete third nerve paralysis. Diagnosis: Locomotor ataxia; fundus showed double choked disk. We thought that there was also an affection of the posterior columns of the cord. The other case was one of double neuro-retinitis. The whole physical examination is negative except the symptoms of falling backward and vomiting without nausea. The case improved on mercury and iodide, though no specific taint could be made out. It was looked over by Drs. Cary, Stockton and Putnam. No definite diagnosis was established.

Dr. Hinkel.—Dr. Krauss's remarks concerning the possible latency of brain abscess, recalls to my mind an interesting though unfortunate case. About ten years ago I operated on a woman past middle life for the relief of nasal polypus. She had been a sufferer from asthma for many years, but otherwise claimed unusually good health and freedom from headache. Polyps had been removed from her nose a number of times, the last operation only a short time before, in another city. To obtain access to the diseased ethnoid cells, I removed the anterior part of the enlarged middle turbinated bone on the left side. After the operation the patient complained of left parietal headache. The second day after, I was called by her physician to see the case with him at her boarding house. Apparently her bed had not been occupied the previous day. She was mentally very dull and lethargic, though she recognised me. She lingered in that condition, with slight fluctuations of temperature for several weeks. At no time was there evidence of inflammation of the nose, though this was carefully looked for. Convulsions and stupor intervened, and Dr. Park trephined the frontal bone just above the left orbit. No pus was found in the basilar portion of the brain, but an abscess of considerable size was evacuated near the vertex. The patient died the next day and the post-mortem revealed a similar abscess in the right frontal lobe and small multiple abscesses throughout the brain. There was thrombosis of the left-middle meningeal artery. There was no evidence of meningeal inflammation above the cribriform plate, and I have always thought that she may have long had a latent brain abscess that unfortunately became acute and infectious after my operation. It is interesting in this connection to mention that intermittent or winking spasm of the eye is often reflex of adenoids, also choreic in character.

In closing Dr. Krauss said: Dr. Blaauw's hysterical ptosis may turn out after all to be ataxia. We must be very careful in our diagnosis between functional and organic disease. We ought to have a train of functional symptoms to back up the diagnosis with. That was the great point Charcot made.. Regarding the length of time a tumor may remain in the brain without causing death, it is impossible to say. I had a case in the Sisters' Hospital in 1892, which had all the symptoms of brain tumor, and we were ready to operate. The mother consulted other physicians who advised her to wait. The symptoms all disappeared and did not recur for five years. Suddenly the same symptoms reappeared and after the diagnosis had again been made, the symptoms in two months disappeared again. Diagnosis first made was abscess of occipital lobe. She has now few symptoms.

Fellows present, 14.

---

### TYPHOID BACILLI FROM THE ROSEOLA.

E. Fraenkel, of Hamburg, (*Zeitsch. Hygiene u. Infectiose Krankheiten*, Bd. xxxiv., Heft. 3), obtained cultures of typhoid bacilli by excising the skin containing the roseola spots and placing them in bouillon. He believes the roseola represent inflammatory spots caused by typhoid bacilli which are hidden in the lymph spaces.—*Med. Age.*

